# Prediction for oxaliplatin‐induced liver injury using patient‐derived liver organoids

**DOI:** 10.1002/cam4.7042

**Published:** 2024-02-24

**Authors:** Kumiko Tatsumi, Hiroshi Wada, Shinichiro Hasegawa, Kei Asukai, Shigenori Nagata, Tomoya Ekawa, Takashi Akazawa, Yu Mizote, Shintaro Okumura, Ryosuke Okamura, Masayuki Ohue, Kazutaka Obama, Hideaki Tahara

**Affiliations:** ^1^ Department of Cancer Drug Discovery and Development, Research Center Osaka International Cancer Institute Osaka Japan; ^2^ Department of Surgery, Graduate School of Medicine Kyoto University Kyoto Japan; ^3^ Department of Gastroenterological Surgery Osaka International Cancer Institute Osaka Japan; ^4^ Department of Diagnostic Pathology and Cytology Osaka International Cancer Institute Osaka Japan; ^5^ Project Division of Cancer Biomolecular Therapy The Institute of Medical Science, The University of Tokyo Tokyo Japan

**Keywords:** liver injury, organoids, oxaliplatin, oxidative stress, sinusoidal obstruction syndrome

## Abstract

**Background:**

Liver injury associated with oxaliplatin (L‐OHP)‐based chemotherapy can significantly impact the treatment outcomes of patients with colorectal cancer liver metastases, especially when combined with surgery. To date, no definitive biomarker that can predict the risk of liver injury has been identified. This study aimed to investigate whether organoids can be used as tools to predict the risk of liver injury.

**Methods:**

We examined the relationship between the clinical signs of L‐OHP‐induced liver injury and the responses of patient‐derived liver organoids *in vitro*. Organoids were established from noncancerous liver tissues obtained from 10 patients who underwent L‐OHP‐based chemotherapy and hepatectomy for colorectal cancer.

**Results:**

Organoids cultured in a galactose differentiation medium, which can activate the mitochondria of organoids, showed sensitivity to L‐OHP cytotoxicity, which was significantly related to clinical liver toxicity induced by L‐OHP treatment. Organoids from patients who presented with a high‐grade liver injury to the L‐OHP regimen showed an obvious increase in mitochondrial superoxide levels and a significant decrease in mitochondrial membrane potential with L‐OHP exposure. L‐OHP‐induced mitochondrial oxidative stress was not observed in the organoids from patients with low‐grade liver injury.

**Conclusions:**

These results suggested that L‐OHP‐induced liver injury may be caused by mitochondrial oxidative damage. Furthermore, patient‐derived liver organoids may be used to assess susceptibility to L‐OHP‐induced liver injury in individual patients.

## Graphical abstract

Liver injury associated with oxaliplatin‐based chemotherapy can significantly impact the treatment outcomes after hepatectomy in patients with colorectal liver metastasis. Our results suggest that oxaliplatin‐induced liver injury in patients is caused by mitochondrial oxidative damage. Furthermore, we provided evidence that individual susceptibility to this liver injury can be assessed using patient‐derived liver organoids with our optimized condition.

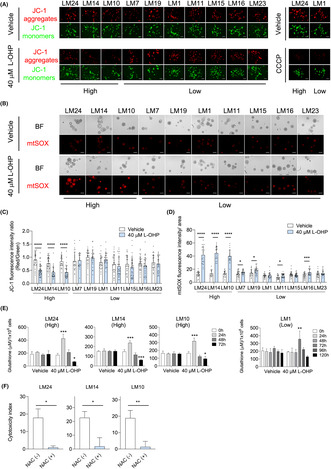

## 1 INTRODUCTION

Oxaliplatin (L‐OHP)‐based regimens are the current standard chemotherapy for metastatic colorectal cancer. Pharmacotherapy with cancer drugs, including L‐OHP, prolongs the overall survival of patients diagnosed with unresectable advanced metastatic colorectal cancer and may also allow curative resection for metastatic lesions.^1^, ^2^ For resectable liver metastases, L‐OHP may be administered as perioperative chemotherapy for patients at high risk of recurrence or incomplete resection. Despite its therapeutic efficacy, liver injury associated with an L‐OHP‐based regimen may lead to morbidity or liver failure following liver resection.^3^, ^4^ Furthermore, precautions for such injury often lead to compromised dosing with an increased risk of early recurrence.^5^ Thus, regimens have to be carefully determined and administered to each patient according to the individual risk of liver injury. However, the management of liver injury has been problematic because of the lack of accurate and reliable biomarkers for predicting the risk of liver injury.

Several biomarkers have been reported to be useful for predicting chemotherapy‐associated side effects in patients with colorectal cancer. Laboratory testing to determine the *UGT1A1* polymorphism helps in predicting the severe side effects such as severe leukopenia or diarrhea by irinotecan.^6^, ^7^, ^8^ The European Medicines Agency recommends the test for dihydropyrimidine dehydrogenase enzyme deficiency, which is related to serious side effects, before starting cancer treatment with the 5‐fluorouracil series.^9^, ^10^ For L‐OHP, Glutathione *S*‐transferase M1 (*GSTM1*)‐null genotype is reported as an independent risk factor for liver injury caused by sinusoidal obstruction syndrome (SOS) in patients with metastatic colorectal cancer.^11^ However, to date, the accuracy of this biomarker remains debated and thus has not been applied in clinical practice. Therefore, new strategies are needed to identify biomarkers, particularly for L‐OHP‐induced liver injury.

Recently, patient‐derived cancer organoids have been considered useful tools to answer clinical questions.^12^ Results of drug sensitivity tests using patient‐derived organoids have been shown to have a significant correlation with the therapeutic outcomes of patients in some models, including L‐OHP,^13^, ^14^, ^15^ suggesting that patient‐derived cancer organoids may be useful in predicting the therapeutic effectiveness of cancer drugs in individual patients. Thus, if patient‐derived liver organoids could also be used to predict L‐OHP‐induced liver injury in treated patients, the risks and benefits of chemotherapy could be assessed before initiating treatment.

Huch et al. reported that liver stem cell‐derived organoids (liver organoids) can be effectively established from a limited amount of liver tissue in the short term. They demonstrated the ability to expand these organoids over an extended period while maintaining genetic stability. Additionally, the liver organoids were successfully induced to undergo functional maturation.^16^ Taking advantage of these characteristics, liver organoids have emerged as valuable tools in the study of hereditary liver diseases, such as α‐1 antitrypsin deficiency.^17^ Nevertheless, the potential utility of patient‐derived liver organoids as predictive models for assessing liver injury caused by chemotherapeutic drugs, including L‐OHP, in actual patients remains largely unexplored.^18^ Liver injury linked to L‐OHP‐based regimens does not manifest uniformly among all patients but rather in certain individuals with varying degrees of severity.^19^, ^20^ This observation implies the presence of individual variations in response to L‐OHP. Therefore, it would be beneficial if patient‐derived liver organoids could be used to predict liver injury caused by L‐OHP. In this study, we found that liver organoids established using our method from non‐cancerous liver tissue of patients could be used to predict L‐OHP‐induced liver injury.

## 2 MATERIALS AND METHODS

### 2.1 Clinical information and specimens

In this study, we enrolled 10 patients who were treated with L‐OHP‐based neoadjuvant chemotherapy and hepatectomy for colorectal cancer liver metastasis at the Osaka International Cancer Institute between April 2019 and February 2020. Clinical information and non‐cancerous liver tissue samples were collected from subjects who provided written informed consent. This study was approved by the ethics committee of Osaka International Cancer Institute (IRB protocol number 18231). The tissue samples were stored in ice‐cold Dulbecco's Modified Eagle's medium/nutrient Mixture F12 (DMEM/F12; Gibco) until processing.

### 2.2 Assessment of liver injury associated with L‐OHP‐based chemotherapy

Liver injury associated with L‐OHP‐based chemotherapy was assessed based on the serum aspartate transaminase (AST), alanine transaminase (ALT), total bilirubin (T‐Bil), and alkaline phosphatase (ALP). The severity of the liver injury was estimated using the Common Terminology Criteria for Adverse Events (CTCAE) v5.0.^21^ The patients were divided into two groups: the high‐grade group, characterized by a grade 1 or higher elevation in both AST and ALT, and the low‐grade group.^4^, ^22^ The L‐OHP‐related injury was defined by changes in the spleen size^23^ and histopathological findings.^24^ Details are provided in Appendix S1.

### 2.3 Liver organoid culture

Liver organoids were established and differentiated into hepatocytes, as described in Appendix S1. Differentiated liver organoids were cultured in a glucose‐based hepatocyte differentiation medium (standard differentiation medium) or galactose‐based hepatocyte differentiation medium (galactose differentiation medium) for in vitro assays. *N*‐acetyl‐L‐cysteine (NAC) was absent during L‐OHP treatment.

### 2.4 Lactate dehydrogenase (LDH) leakage assay

Cytotoxicity was assessed by measuring LDH released from the cytoplasm into the culture medium upon loss of cytoplasmic membrane integrity.^25^ Cells were seeded at a density of 1500 cells into a 96‐well plate in triplicate, cultured in expansion medium supplemented with 25 ng/mL human BMP‐7 (R&D Systems, 354‐BP‐010) for 3–5 days, and then cultured with differentiation medium until the LDH leakage assay. After hepatocyte differentiation, the organoids were cultured in a differentiation medium (NAC‐free) containing rotenone (TCl, R0090) or L‐OHP (TCl, O0372). For the rotenone treatment, the supernatant was collected 24 h later. For the L‐OHP treatment, the supernatant was collected and replaced with a fresh drug‐containing medium every 24 h for up to 72 h. The L‐OHP doses were set from the maximum plasma concentration (*C*_max_) during clinical administration to doses of up to four times higher than *C*_max_. LDH activity was measured using an LDH Cytotoxicity Assay Kit (Nacalai Tesque) according to the manufacturer's protocol. Absorbance was measured at 490 nm using a Multimode Microplate Reader Infinite M200 Plex (Tecan). LDH release and the cytotoxicity index were calculated according to the following formula^26^:

$$\mathrm{LDH}\;\text{release}\;\left(\%\right)=\frac{\text{experimental release}\;}{\text{maximum release}}\times 100$$

$$\begin{array}{c} \text{Cytotoxicity index}=\frac{\left(\text{experimental release}-\text{spontaneous release}\right)}{\left(\text{maximum release}-\text{spontaneous release}\right)}\\ \;\times 100 \end{array}$$

Spontaneous release represents LDH released from untreated cells, and maximum release represents LDH released from cells treated with lysis buffer. Additional information for in vitro experiments is described in Appendix S1.

### 2.5 Statistical analyses

Statistical differences for single comparisons were evaluated using a two‐sided Welch's *t*‐test or the non‐parametric Mann–Whitney *U* test. Statistical differences for multiple comparisons were evaluated using a one‐way ANOVA with Dunnett's test. All statistical tests were performed using GraphPad PRISM 8. *p* < 0.05 was considered statistically significant.

## 3 RESULTS

### 3.1 Assessment of liver injury in patients receiving L‐OHP‐based chemotherapy

To investigate individual differences in their susceptibility to L‐OHP‐induced liver injury, 10 patients who underwent liver resection after receiving L‐OHP chemotherapy were recruited for analysis. The clinical information of the 10 patients is presented in Table 1. Among the 10 patients, three patients (LM24, LM14, and LM10) who developed grade 1 or higher elevation of both AST and ALT in the blood test data were classified into the high‐grade liver injury group. The remaining seven patients (LM7, LM19, LM1, LM11, LM15, LM16, and LM23) who developed grade 1 elevation in either AST or ALT or no elevation in both AST and ALT levels were classified into the low‐grade liver injury group.

**TABLE 1:** Clinical information of subjects including chemotherapy regimens and liver injury.

Liver Injury	Patient I.D.	Age	Sex	L‐OHP based chemotherapy		CTCAE grade				Spleen size (post/pre)
				Regimen	Cycles	AST	ALT	T‐Bil	ALP	
High	LM24	49	Male	XELOX	8	3	3	1	2	1.6
High	LM14	77	Female	mFOLFOX6	6	1	1	–	–	1.3
High	LM10	37	Male	XELOX	5	1	1	–	–	2.3
Low	LM7	61	Male	XELOX	3	1	–	–	–	0.9
Low	LM19	58	Male	FOLFOXIRI	10	–	1	–	1	1.0
Low	LM1	67	Male	mFOLFOX6	8	–	–	–	–	1.0
Low	LM11	38	Female	mFOLFOX6	5	–	–	–	–	0.9
Low	LM15	58	Male	XELOX	8	–	–	–	–	1.0
Low	LM16	70	Female	XELOX	4	–	–	–	–	1.1
Low	LM23	70	Male	mFOLFOX6	8	–	–	–	–	1.0

*Note*: Spleen size (post/pre) = spleen volume after chemotherapy/spleen volume before chemotherapy. Spleen volume was calculated by multiplying the length, thickness, and width.

Abbreviations: ALP, alkaline phosphatase; ALT, alanine aminotransferase; AST, aspartate aminotransferase; FOLFOXIRI, leucovorin, 5‐fluorouracil, irinotecan, and L‐OHP; T‐Bil, total bilirubin; XELOX, capecitabine and L‐OHP; mFOLFOX6, leucovorin, 5‐fluorouracil, and L‐OHP.

**FIGURE 1:**
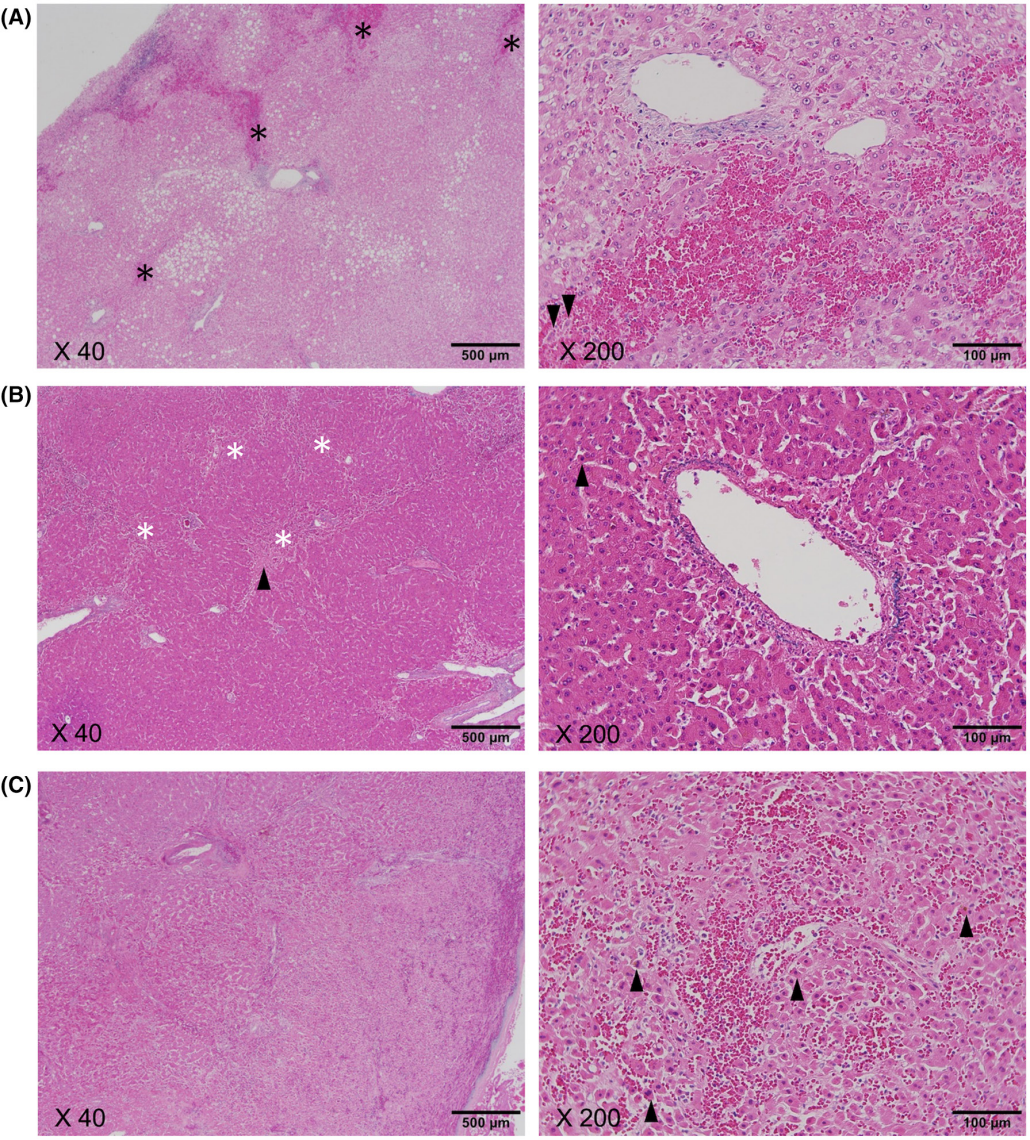
Histology of liver tissues in the high‐grade liver injury group. (A–C) Representative Victoria‐Blue (VB)‐H&E staining images of non‐cancerous liver tissues treated with L‐OHP‐based chemotherapy. Low‐ and high‐power fields are shown in the left and right panels, respectively. (A) Panel of LM10. Sinusoidal congestion (black asterisks) was observed (left panel). Moderate sinusoidal dilatation around the central vein, hepatocyte atrophy, hepatocyte degeneration (arrowheads), and congestion were observed (right panel). (B) Panel of LM24. Sinusoidal dilatation and hepatocyte atrophy (white asterisks), and hepatocyte degeneration (arrowhead) were observed (left panel). Mild sinusoidal dilatation around the central vein and hepatocyte degeneration (arrowhead) were observed (right panel). (C) Panel of LM14. Lobular lesions with sinusoidal dilatation are observed (left panel). Moderate to severe sinusoidal dilatation around the central vein, disordered hepatocyte arrangement, and hepatocyte degeneration (arrowheads) were observed (right panel).

We examined the increase in spleen size which is a clinical characteristic of L‐OHP‐induced hepatotoxicity.^23^ An increase in spleen size following L‐OHP‐based chemotherapy was observed in the high‐grade group, while such an increase was not prominently observed in the low‐grade group (Table 1). Furthermore, a histopathological examination was performed to estimate the presence of L‐OHP‐related liver injury. In the liver tissues of three patients in the high‐grade group, sinusoidal dilation, centrilobular/venular fibrosis (except for LM14), nodular transformation, and hepatocellular damage were observed (Figure 1A,B,C, Table S1). These pathological findings are compatible with the characteristics of the liver with L‐OHP‐induced hepatotoxicity.^24^ In contrast, in the liver tissues of seven patients in the low‐grade group, histological changes were relatively mild (Figure S1, Table S1). Thus, the histopathological changes observed in the liver tissues were consistent with the grade of liver injury in the blood test data (Table 1).

### 3.2 Liver organoids cultured in standard differentiation medium do not show cytotoxic reactions with L‐OHP treatment

In all 10 patients, organoids were successfully established from non‐cancerous liver tissues within 2 weeks. Organoids cultured in an expansion medium consisted of a single‐layered epithelium, whereas those cultured in a differentiation medium changed to a stratified polygonal epithelium (Figure S2A,B). Compared to the organoids in the expansion medium, the organoids in the differentiation medium expressed the adult stem cell marker (*LGR5*) at a lower level and the mature hepatocyte markers (*HNF4A*, *ALB*, and *CYP3A4*) at higher levels (Figure S2C). These characteristics were similar to those reported for liver organoids by Huch et al.^16^

To develop an in vitro assay to predict the likelihood of liver injury related to L‐OHP in patients, we examined the cytotoxic effects of L‐OHP on liver organoids cultured in a standard differentiation medium. Even with repeated doses of 40 μM L‐OHP, which is a quadruple dose of *C*_max_ during clinical administration, the average levels of LDH release in the high‐ and low‐grade groups were 6.9 ± 1.9% and 6.5 ± 3.2%, respectively (Figure S3A). Furthermore, the levels of L‐OHP‐induced cytotoxicity were not significantly different between the two groups at any concentration of L‐OHP (Figure S3B). These results suggest that in vitro assays under these culture conditions may not be sensitive enough to predict the likelihood of liver injury related to L‐OHP in these patients.

It has been reported that L‐OHP leads to the generation of reactive oxygen species (ROS) from the mitochondria and can result in liver injury.^27^, ^28^, ^29^ Therefore, it was considered that the cytotoxic effects of L‐OHP cannot be fully observed in cells with insufficient mitochondrial activity. Thus, we performed a JC‐1 assay to detect mitochondrial membrane potential as a parameter of mitochondrial condition. Red fluorescence represents JC‐1 aggregates appearing at high membrane potentials, indicating healthy mitochondria. As shown in Figures S3C and S4, liver organoids from all 10 patients showed green fluorescence (JC‐1 monomers), whereas red fluorescence (JC‐1 aggregates) was not observed. These results suggest that the mitochondria were inactive under these culture conditions. Therefore, the cytotoxic effects of L‐OHP could not be accurately estimated using the assay conditions described above.

### 3.3 Liver organoids cultured in galactose differentiation medium show sensitivity to L‐OHP cytotoxicity correlating with the grade of L‐OHP liver injury in patients

In a previous study, it was shown that isolated primary mature hepatocytes switch energy production from mitochondrial oxidative phosphorylation to glycolysis in a high glucose medium.^30^ Given that the standard differentiation medium contains high glucose, liver organoids in such conditions may also rely on glycolysis as their major source of energy production. We hypothesized that this might be the reason why mitochondria‐related toxicity was not observed, as described above. HepG2 cells, which are widely used for mitochondrial toxicity studies, grown in the presence of galactose instead of glucose, are reportedly forced to shift most of their energy production to mitochondrial oxidative phosphorylation and exhibit sensitivity to mitochondrial toxins.^31^ In this study, we investigated whether the medium in which glucose was replaced with galactose could be applied to liver organoids and used for cytotoxicity assays.

**FIGURE 2:**
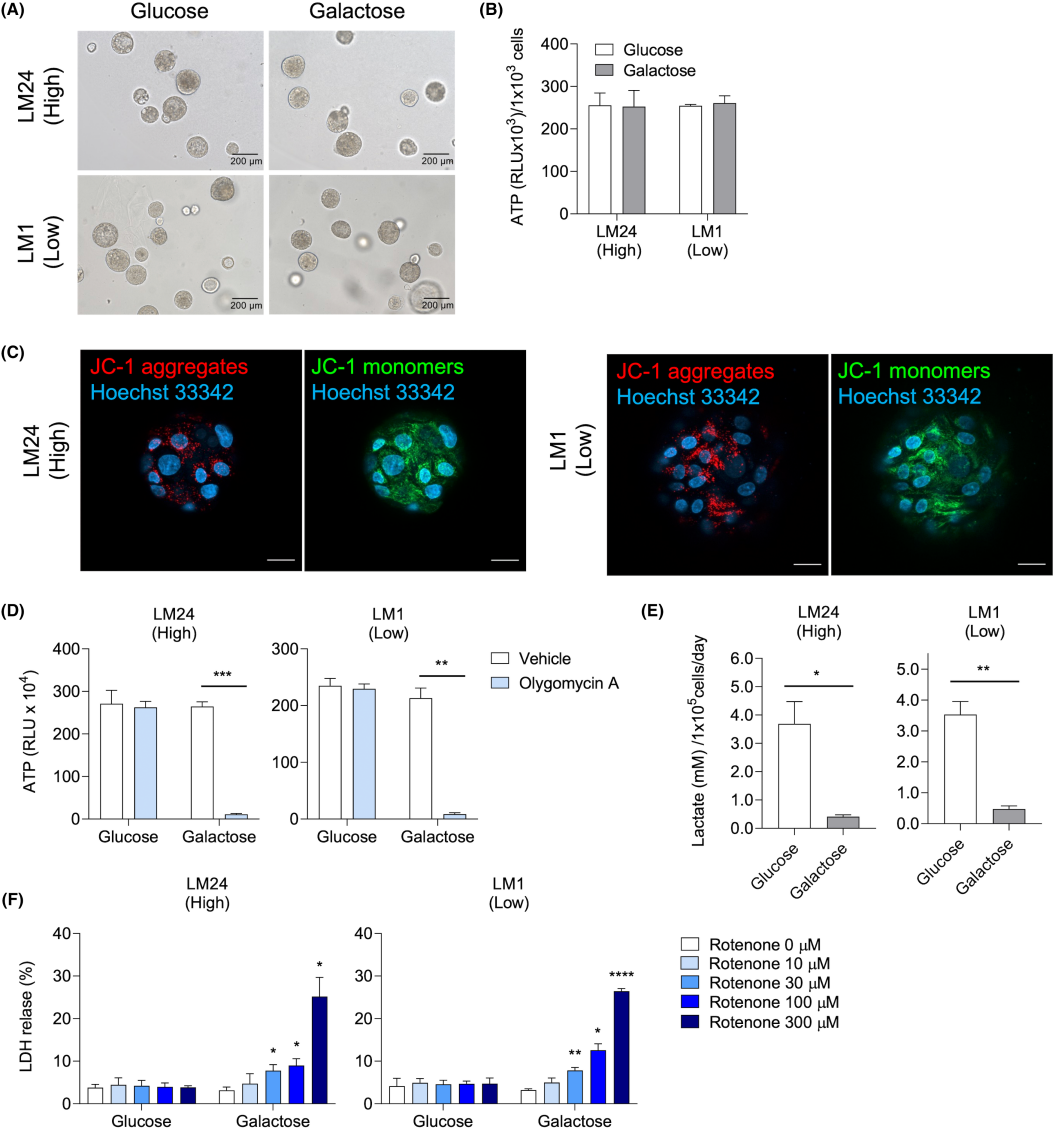
Differences in mitochondrial activity of liver organoids between standard differentiation medium and galactose differentiation medium. (A–F) Liver organoids of LM24 (high‐grade group) and LM1 (low‐grade group) were cultured in standard (glucose) or galactose differentiation medium for 8–11 days. (A) Representative brightfield images of liver organoids. (B) Cell viability was evaluated by measuring intracellular ATP levels. (C) Mitochondrial condition was evaluated with a JC‐1 probe. Representative images of JC‐1‐stained liver organoids are shown. Red, JC‐1 aggregates; green, JC‐1 monomers; blue, Hoechst 33342 (nuclei). Scale bar, 20 μm. (D) Intracellular ATP levels of liver organoids cultured with vehicle (0.3% DMSO) or 5 μM oligomycin A for 2 h. (E) The amount of lactate in the medium after 24 h culture. (F) LDH release from liver organoids treated with rotenone at the indicated concentrations. These assays were performed in duplicate and representative data were shown. Bars indicate mean ± SD. Statical significance was determined with a two‐sided Welch's *t*‐test (D, E) or ANOVA followed by Dunnett's test (compared with 0 μM of rotenone) (F). *p < 0.05, **p < 0.01, ***p < 0.001, **** *p* < 0.0001.

Among the 10 patients, LM24 developed the highest elevation in AST, ALT, T‐Bil, and ALP, whereas LM1 developed no elevation in these blood test items associated with liver damage (Table 1). LM24 and LM1 were used as representatives of the high‐ and low‐grade groups, respectively. LM24 and LM1 cultured in galactose differentiation medium maintained similar shapes and produced similar amounts of ATP as those cultured in standard differentiation medium (Figure 2A,B). Unlike in the standard differentiation medium, the mitochondria of the organoids were active in the galactose differentiation medium (Figure S3C, S4, 2C). Since there was no apparent difference in the basal mitochondrial activity between the high‐ and low‐grade groups, we proceeded with further analysis.

Next, we examined the dependence of liver organoids on mitochondrial oxidative phosphorylation during energy production. Intracellular ATP levels were measured in the presence of oligomycin A, an inhibitor of mitochondrial ATP synthase.^32^ In the standard differentiation medium, oligomycin A had no effect, whereas oligomycin A resulted in a dramatic decrease of ATP content in the galactose differentiation medium (Figure 2D). In addition, LM24 and LM1 organoids cultured in galactose differentiation medium produced significantly less lactic acid, a glycolysis metabolite, than those cultured in standard differentiation medium (Figure 2E). These results strongly suggest that the galactose differentiation medium enables energy production in liver organoids through mitochondrial oxidative phosphorylation instead of glycolysis. Furthermore, to confirm the sensitivity to mitochondrial toxins, we treated LM24 and LM1 organoids with rotenone. Rotenone is an inhibitor of mitochondrial electron transport chain complex I and leads to the restriction of ATP synthesis.^33^ Both LM24 and LM1 exhibited no significant increase in LDH leakage when cultured in the standard differentiation medium. However, when exposed to a galactose differentiation medium, both organoids demonstrated a concentration‐dependent elevation in LDH leakage (Figure 2F). These results indicated that mitochondrial injury‐induced cytotoxicity could be examined if the galactose differentiation medium was used for the culture of liver organoids.

**FIGURE 3:**
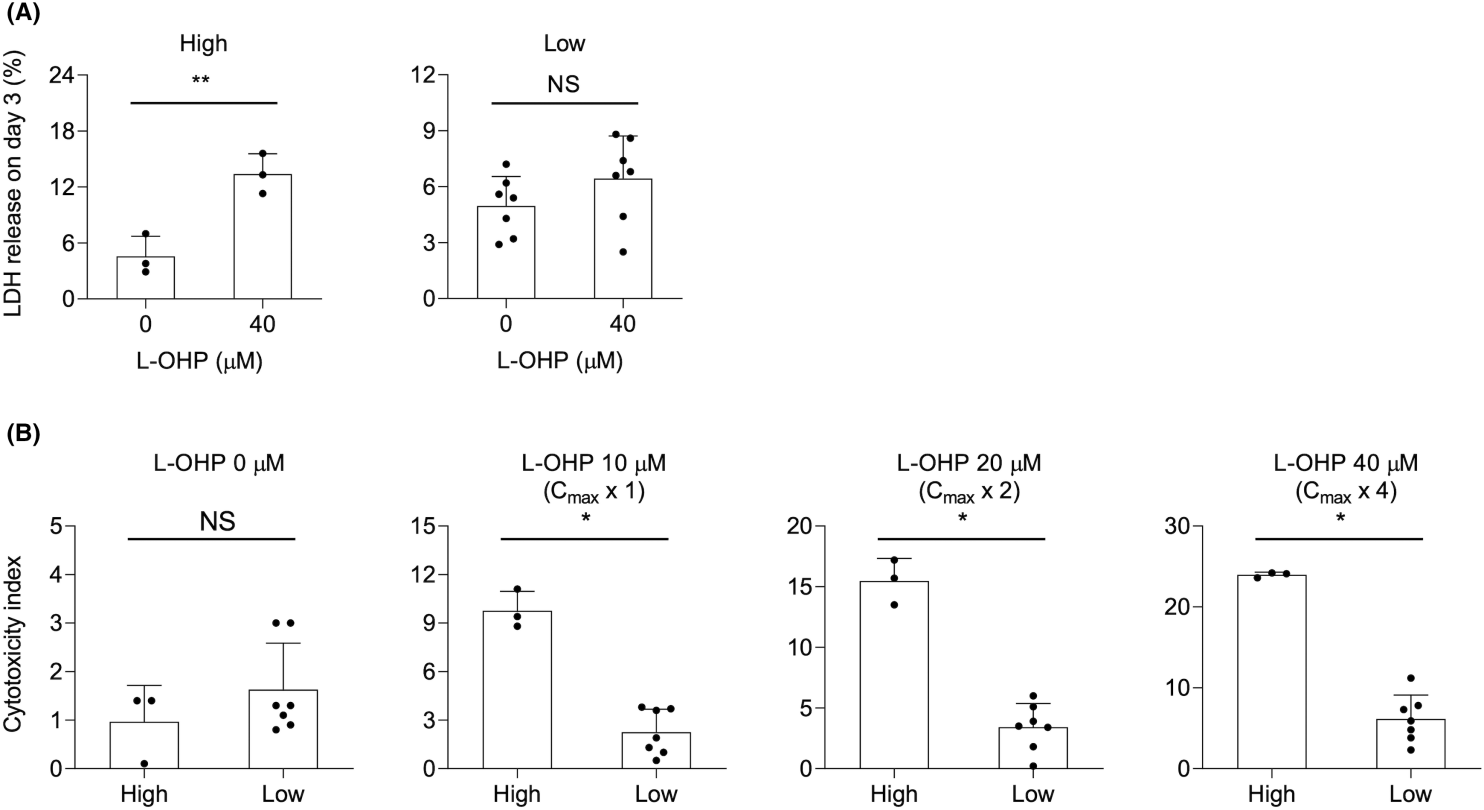
L‐OHP‐induced cytotoxicity in liver organoids cultured in galactose differentiation medium. (A and B) Liver organoids cultured for 5 days in standard differentiation medium followed by 3 days in galactose differentiation medium were subjected to repeated doses of L‐OHP. (A) LDH release from liver organoids on day 3 of repeated doses of L‐OHP. (B) Cytotoxicity index of liver organoids treated with repeated doses of L‐OHP at the indicated concentrations for 72 h. Each dot indicates independent patients in the high‐ or low‐grade groups. Bars indicate mean ± SD. Statical significance was determined with a two‐sided Welch's *t*‐test (A) or two‐sided Mann–Whitney *U* test (B). *p < 0.05, **p < 0.01. NS, Not Significant.

Liver organoids from each patient were cultured in a galactose differentiation medium and tested for L‐OHP cytotoxicity. With a repeated maximum dose of 40 μM L‐OHP, LDH release levels for the high‐ and low‐grade groups were 13.4 ± 2.1% and 6.5 ± 2.3%, respectively (Figure 3A). Organoids in the high‐grade group showed significantly higher levels of LDH release, which were not observed in cultures in the standard differentiation medium (Figure S3A, 3A). Moreover, the organoids in the high‐grade group showed significantly higher cytotoxicity indices after L‐OHP treatment than those in the low‐grade group (Figure 3B).

### 3.4 L‐OHP‐induced liver toxicity is caused by mitochondrial oxidative damage

**FIGURE 4:**
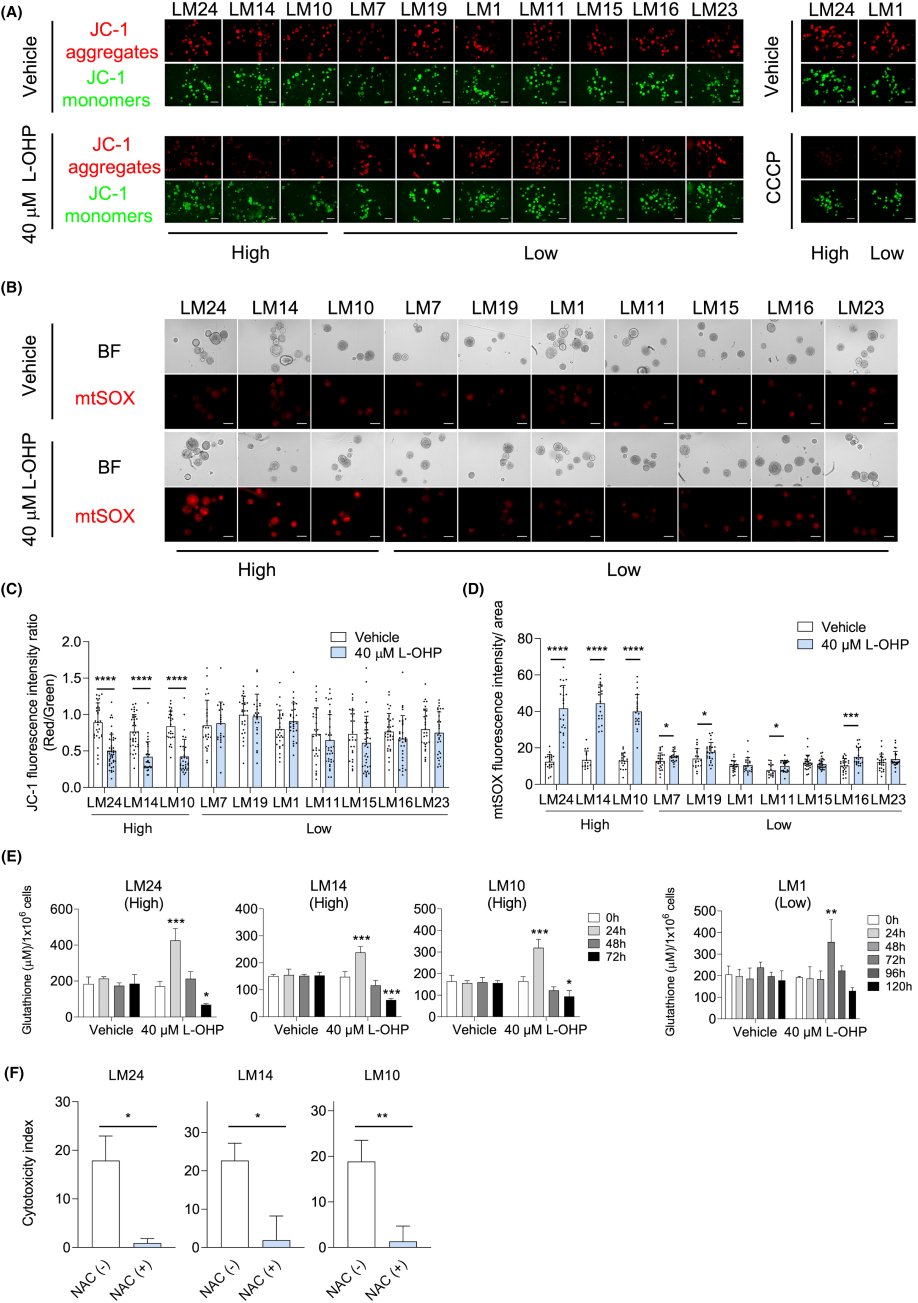
L‐OHP‐induced mitochondrial oxidative stress in liver organoids in each patient. (A‐F) Liver organoids cultured for 5 days in standard differentiation medium followed by 3 days in galactose differentiation medium were subjected to a single dose or repeated doses of L‐OHP. (A) Mitochondrial condition was evaluated with a JC‐1 probe after the repeated doses of L‐OHP for 48 h or CCCP treatment for 1.5 h. Representative images of JC‐1‐stained liver organoids are shown. Red, JC‐1 aggregates; green, JC‐1 monomers. Scale bar, 500 μm. (B) Mitochondrial superoxide (O2^●‐^) generation was visualized with mtSOX Deep Red probe after 0 and 40 μM L‐OHP treatment for 2 h. Representative images of the liver organoids are shown. Red, O2^●‐^. BF, brightfield. Scale bar, 200 μm. (C) Fluorescence intensity ratio (red/green) of (A). Each dot indicates an organoid. (D) Fluorescence intensity per area of (B). Each dot indicates an organoid. (E) The amount of intracellular total GSH at each time point of repeated doses administered every 24 h, extending up to either 72 h or 120 h. (F) Cytotoxicity index of liver organoid with repeated doses of 40 μM L‐OHP ± 2 mM NAC for 72 h. These assays were performed in duplicates and representative data were shown. Bars indicate mean ± SD. Statical significance was determined with a two‐sided Welch's *t*‐test (C, D, F) or ANOVA followed by Dunnett's test (compared with t = 0 h) (E). *p < 0.05, **p < 0.01, ***p < 0.001, ****p < 0.0001.

Mitochondrial conditions were evaluated using the JC‐1 assay. The liver organoids showed a decrease in red fluorescence with treatment of CCCP, a mitochondrial oxidative phosphorylation uncoupler, indicating that the JC‐1 assay was working (Figure 4A). The liver organoids of the low‐grade group showed no significant changes in the fluorescence intensity ratio (red [JC‐1 aggregates]/green [JC‐1 monomers]) with L‐OHP treatment compared to vehicle treatment. However, liver organoids of the high‐grade group showed a significant decrease in the fluorescence intensity ratio (Figure 4A,C). To investigate the generation of mitochondrial ROS by L‐OHP treatment, mitochondrial superoxide (O_2_^●‐^) after 2 h of L‐OHP treatment was examined using a mtSOX Deep Red probe emitting red fluorescence. In liver organoids from multiple patients, red fluorescence was significantly increased with L‐OHP treatment compared to vehicle treatment, exhibiting spontaneous generation. Importantly, the high‐grade group showed an obvious increase in red fluorescence compared with the low‐grade group (Figure 4B,D). In the high‐grade group, the decreased mitochondrial activity was consistent with increased superoxide generation. Moreover, within the high‐grade group, there was an observed increase in the total intracellular glutathione (GSH) levels 24 h following L‐OHP treatment. However, these levels subsequently decreased over time. LM1, which was used as a representative for the low‐grade group, showed increasing GSH levels with the repeated administration of L‐OHP when compared to the high‐grade group (Figure 4E). GSH is a well‐recognized direct antioxidant that is widely involved in the cellular removal of H_2_O_2_ and other hydroperoxides.^34^, ^35^ When cells are under oxidative stress, GSH biosynthesis is promoted by free radicals.^36^ Our results suggest that GSH is transiently biosynthetically increased by L‐OHP treatment and then consumed. NAC acts as a direct and indirect antioxidant by upregulating antioxidant enzymes such as Mn‐SOD, Cu/Zn‐SOD, glutathione peroxidase (GSH‐Px), and catalase (CAT), or by acting as a GSH precursor.^35^, ^37^ Cytotoxicity was suppressed by the concomitant use of high‐dose NAC (Figure 4F). Thus, L‐OHP‐induced cytotoxicity in the liver organoids may be attributed to mitochondrial oxidative damage.

**FIGURE 5:**
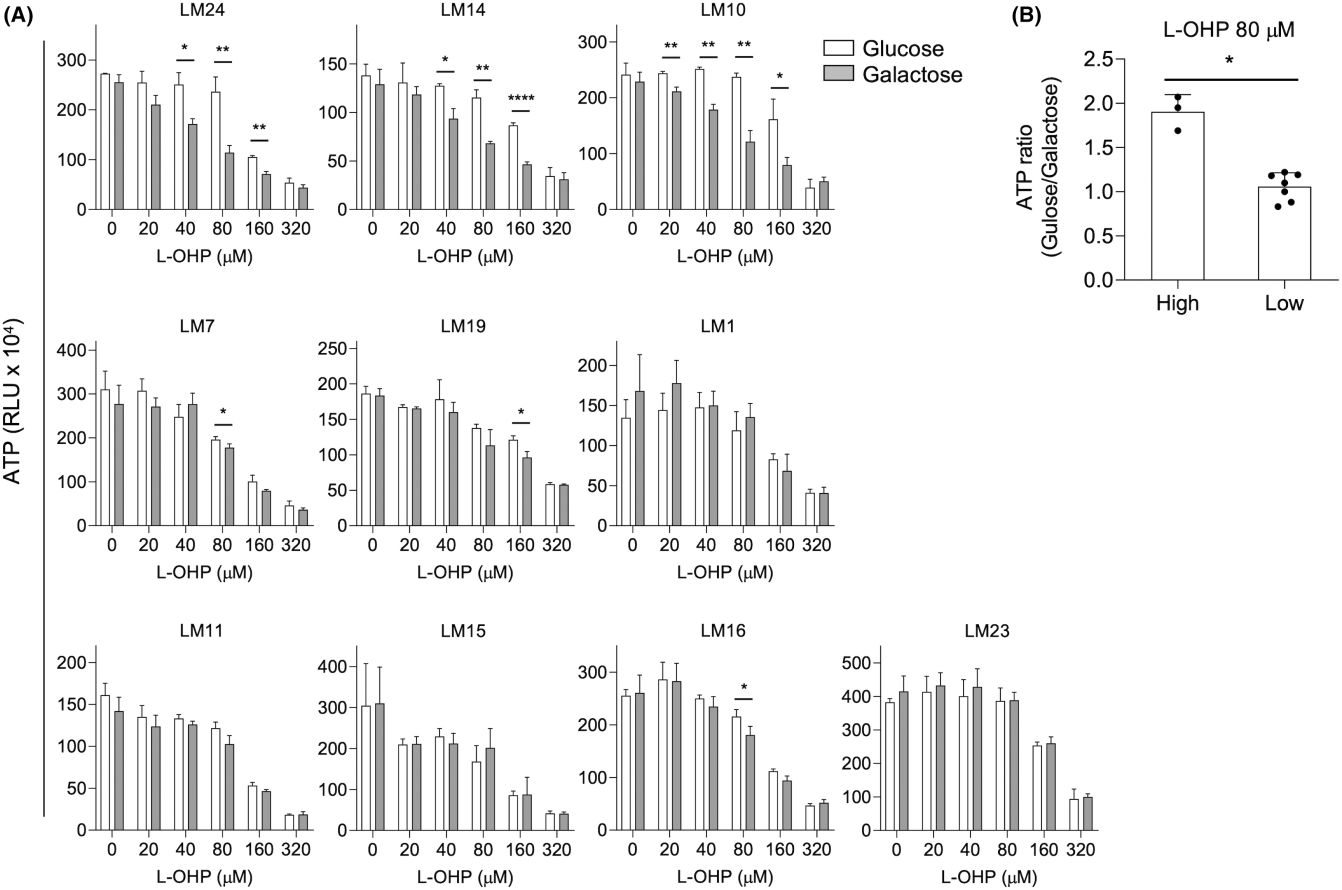
A simple test method to assess the risk of hepatic mitochondrial oxidative damage. (A) Intracellular ATP levels in liver organoids cultured in standard (glucose) or galactose differentiation medium with a single dose of L‐OHP at the indicated concentrations for 72 h. (B) The ratio of intracellular ATP (glucose/galactose) in liver organoids at 80 μM L‐OHP is presented in (A). Each dot indicates independent patients in the high‐grade group or the low‐grade group. Bars indicate mean ± SD. Statical significance was determined with a two‐sided Welch's *t*‐test (A) or two‐sided Mann–Whitney *U* test (B). *p < 0.05. **p < 0.01. ****p < 0.0001.

Finally, to develop a simple and clinically applicable test method for the assessment of mitochondrial oxidative damage in patients, we examined the intracellular ATP levels in liver organoids cultured under different conditions. Liver organoids from each patient were cultured in standard or galactose differentiation media and treated with a single dose of L‐OHP for 72 h. Intracellular ATP levels in organoids from the high‐grade group showed significant differences between the two types of media in the range of 20–160 μM L‐OHP at multiple points. At an L‐OHP concentration of 80 μM, the liver organoids from the high‐grade group showed significantly higher ATP ratios than those of liver organoids from the low‐grade group (Figure 5A,B).

## 4 DISCUSSION

While surgical resection of colorectal cancer liver metastasis can prolong survival time,^38^ it is important to note that the recurrence of liver metastasis is a common occurrence, with rates ranging between 45% and 70%.^39^, ^40^ In such situations, the majority of these patients are typically treated with chemotherapy.^2^, ^39^ We have proposed an in vitro examination method to assess the susceptibility of L‐OHP‐induced liver injury using patient‐derived liver organoids. This assessment could prove highly beneficial for selecting appropriate treatment options, such as chemotherapy with or without L‐OHP or treatment prior to surgical resection in the event of recurrence. The advantage of this approach is that the assessment can be completed within approximately 1 month after liver resection. This timeframe allows for an ample window to assess the susceptibility of L‐OHP‐induced liver injury and devise a treatment strategy before recurrence is confirmed.

Multiple investigators have examined the usefulness of patient‐derived liver organoids for evaluating drug potency in vitro. However, we found that the mitochondrial functions of liver organoids could not be fully examined if the liver organoids were cultured in commonly used maturation media. To improve this status, we developed an appropriate culture condition and examined the mechanism of L‐OHP toxicity, which has been implicated in oxidative stress in mouse models.^28^

In our study, the patients categorized into the high‐grade liver injury group presented clinical symptoms characteristic of L‐OHP‐induced liver injury with relatively mild elevations in AST and ALT, which suggests the possibility of severe L‐OHP‐induced liver injury.^4^, ^22^ The patients categorized in the low‐grade liver injury group appeared to have no or undetectable L‐OHP‐induced liver injury since they presented no clinical symptoms characteristic of L‐OHP. Thus, the examination of liver organoids derived from these patients would provide us with good opportunities to examine the differences in the characteristics of patient‐derived liver organoids by utilizing their clinical information.

The mechanisms underlying L‐OHP‐induced liver toxicity have been previously studied in animal models by multiple researchers. Robinson et al. showed a reduction in total liver GSH with FOLFOX‐induced SOS and upregulation of genes implicated in oxidative stress in a mouse model.^28^ Lin et al. and Lu et al. also studied L‐OHP‐induced liver toxicity using mouse models and found increased levels of malondialdehyde and GSH or ROS and decreased levels of SOD and GSH‐Px in the liver with L‐OHP treatment.^41^, ^42^ These reports demonstrated that oxidative stress plays an important role in L‐OHP‐induced liver toxicity in vivo. Additionally, in a study by Tabassum et al., liver mitochondria extracted from rats were utilized, revealing that treatment with L‐OHP led to heightened oxidative stress markers as well as elevated mitochondrial Mn‐SOD activities. Furthermore, the study observed a decrease in GSH levels, suggesting that mitochondria are susceptible targets for L‐OHP‐induced toxicity.^29^ These reports collectively suggest that the toxic response to L‐OHP may be caused by the oxidative stress on mitochondria.

L‐OHP treatment may damage liver sinusoidal epithelial cells (LSECs) and disrupt the hepatocyte plate, resulting in sinusoidal injury and impaired hepatic circulation.^24^, ^28^, ^43^ Thus, in vitro assays using primary LSECs or primary hepatocytes from individual patients may provide useful information on liver toxicity if the mitochondrial oxidative stress induced by L‐OHP is properly examined. However, both primary cell types obtained from individual patients cannot be used since they already have mitochondrial membrane damage due to cryopreservation.^44^, ^45^ In contrast, the mitochondrial membrane and functions of liver organoids were found to be preserved, as shown in our current study. And they were able to survive in a galactose medium which would force the cultured cells to shift their major source of energy production from glycolysis to mitochondrial oxidative phosphorylation. Changes in the energy metabolism of liver organoids due to different sugar sources have not been previously studied. Galactose undergoes conversion into glucose‐1‐phosphate through the Leloir pathway, subsequently entering both glycolysis and oxidative phosphorylation.^46^ In situations where the sole sugar source is galactose, this conversion process consumes ATP, leading to a reduction in the net ATP production from glycolysis. Consequently, cells must rely more heavily on oxidative phosphorylation than glycolysis to generate the ATP required for survival.^47^, ^48^, ^49^ L‐OHP‐induced cytotoxicity can probably only be reliably assessable under conditions where the energy metabolism of cells resembles that of in vivo liver cells, which predominantly rely on oxidative phosphorylation for ATP production.

A novel finding in our study was the correlation observed between L‐OHP‐induced cytotoxicity in patient‐derived liver organoids, attributed to mitochondrial oxidative damage, and the clinical severity of L‐OHP‐induced liver injury. The factors that modulate susceptibility to L‐OHP‐induced oxidative damage in the liver remain unclear. Based on our results, we discuss the possibility of these underlying factors. In our study, liver organoids derived from the high‐grade group showed an obvious increase in O_2_^●‐^ levels early after L‐OHP treatment. O_2_^●‐^ forms peroxynitrite (ONOO^−^) and hydroxyl radicals (^●^OH), which are the most reactive oxygen species that interact with lipids, DNA, and proteins, causing the loss of mitochondrial membrane integrity.^50^, ^51^ Therefore, enzyme activities involved in regulating O_2_^●‐^ levels and subsequent antioxidant processes may play an important role in mitochondrial oxidative stress management. For instance, genetic polymorphisms of Mn‐SOD, an enzyme that eliminates O_2_^●‐^, and GSH‐Px, have been reported to affect their enzymatic activities and are involved in drug‐induced liver injury susceptibility.^52^, ^53^ Intracellular concentrations of L‐OHP may also vary among individuals. For instance, the glutathione‐S‐transferase (GST) enzyme mediates the conjugation of GSH with L‐OHP, and this conjugate is subsequently excreted from the cell^54^; however, it has been reported that the *GSTM1*/*GSTT1*‐null genotype loses GST activity.^55^ Organic cationic transporter 1 plays an important role in L‐OHP uptake,^56^ and these variants are known to reduce transport activity.^57^ Further examination of these possibilities will enhance our understanding about the individual susceptibilities to L‐OHP‐induced liver injury.

The compounds of interest can be categorized into mitochondrial and non‐mitochondrial toxicants using the assay supplied with two types of sugar, glucose, and galactose.^58^ Herein, we show the usefulness of a simple and clinically applicable test method using liver organoids cultured in two types of medium and their ATP ratios to assess the risk of hepatic mitochondrial oxidative damage. Using this method, the ATP ratios in the 80 μM L‐OHP treatment were clearly different between the high‐ and low‐grade groups. We examined liver organoids derived from resected liver tissues of patients who underwent L‐OHP‐based chemotherapy. While our observations revealed preserved mitochondrial membrane integrity and functions in the liver organoids across all patients, it is important to note that we cannot definitively exclude the possibility of our results being influenced by preceding mitochondrial damage caused by L‐OHP. Therefore, prospective studies using liver organoids derived from chemotherapy‐naive patients are required to confirm our findings. Our results warrant further examination with a larger number of patients.

In conclusion, our results suggest that L‐OHP‐induced liver injury is caused by mitochondrial oxidative damage. Furthermore, we provided evidence that individual susceptibility to L‐OHP‐induced liver injury can be assessed using patient‐derived liver organoids to provide useful information for planning chemotherapy. This in vitro test method might also be useful for predicting liver toxicities in individual patients receiving other cancer drugs that cause liver mitochondrial damage, including sorafenib and regorafenib,^59^ used for hepatocellular carcinoma.

## AUTHOR CONTRIBUTIONS

**Kumiko Tatsumi:** Conceptualization (lead); data curation (equal); formal analysis (lead); funding acquisition (supporting); investigation (lead); methodology (lead); project administration (equal); visualization (lead); writing – original draft (lead); writing – review and editing (lead). **Hiroshi Wada:** Methodology (supporting); resources (lead); writing – review and editing (equal). **Shinichiro Hasegawa:** Methodology (supporting); resources (lead); writing – review and editing (equal). **Kei Asukai:** Methodology (supporting); resources (lead); writing – review and editing (equal). **Shigenori Nagata:** Investigation (supporting); methodology (supporting); resources (lead); visualization (supporting); writing – review and editing (equal). **Tomoya Ekawa:** Formal analysis (supporting); investigation (supporting); writing – review and editing (equal). **Takashi Akazawa:** Data curation (equal); formal analysis (supporting); investigation (supporting); writing – review and editing (equal). **Yu Mizote:** Data curation (equal); formal analysis (supporting); investigation (supporting); writing – review and editing (equal). **Shintaro Okumura:** Conceptualization (lead); methodology (lead); project administration (equal); supervision (lead); writing – original draft (lead); writing – review and editing (lead). **Ryosuke Okamura:** Conceptualization (supporting); project administration (equal); supervision (supporting); writing – original draft (supporting); writing – review and editing (equal). **Masayuki Ohue:** Resources (supporting); writing – review and editing (equal). **Kazutaka Obama:** Conceptualization (supporting); project administration (equal); supervision (lead); writing – review and editing (equal). **Hideaki Tahara:** Conceptualization (lead); funding acquisition (lead); project administration (equal); supervision (lead); writing – original draft (lead); writing – review and editing (equal).

## FUNDING INFORMATION

This work was supported by funding from the Osaka International Cancer Institute and partly by the Project for Research on Development of New Drugs (grant number 22ak0101187h0101) from the Japan Agency for Medical Research and Development.

## CONFLICT OF INTEREST STATEMENT

The authors have no conflict of interest.

## ETHICS STATEMENT

Approval of the research protocol by an Institutional Review Board: The present study was approved by the ethics committee of the Osaka International Cancer Institute (IRB protocol number: 18231). This study was conducted in accordance with the principles of the Declaration of Helsinki.

## CONSENT

Written informed consent was received from all patients.

Registry and the Registration No. of the study/trial: N/A.

Animal Studies: N/A.

## Supporting information


Appendix S1.

Figures S1–S4.

Table S1.

## Data Availability

The data that support the findings of this study are available from the corresponding author upon reasonable request.
