# Supplementary material for: Prediction for oxaliplatin‐induced liver injury using patient‐derived liver organoids
Source: Cancer Med. 2024 Feb 24;13(3):e7042. doi: 10.1002/cam4.7042 (PMC10891453; doi:10.1002/cam4.7042)
Supplement: Supplementary file 1 — Appendix S1. [file CAM4-13-e7042-s003.docx]

Supporting Information

**Appendix S1. Supplementary Materials and Methods**

**Change in spleen size**

The spleen size was determined by measuring computed tomography (CT) images using Centricity Enterprise Web software (version 3.0; GE Medical Systems, USA). According to a report by Prassopoulos *et al*.,^1^ the maximum width (W) of the spleen, splenic height or length (L), and thickness at the hilum (Th) were measured, and the spleen volume was calculated using the following formula:

Spleen volume (cm^3^) = 30 + 0.58 (W × L × Th)

Changes in splenic size were determined by comparing the splenic volumes before and after L-OHP-based chemotherapy.

**Histopathologic examination**

Archival slides stained with Victoria Blue (VB)-H&E were available for all patients. Slides for evaluation were selected from non-neoplastic liver parenchymal sites at least 20 mm from the tumor. Histological evaluation was performed by a pathologist (S.N.) who was blinded to the patient’s clinical and laboratory findings. The pathological findings of sinusoidal dilatation and, depending on severity, hepatocellular damage, centrilobular/venular fibrosis, nodular transformation, and peliosis have been reported in L-OHP-induced hepatotoxicity.^2-5^ Histopathological features were classified and graded according to the criteria of Rubbia-Brandt *et al*.^3^ Based on the criteria, sinusoidal dilation was classified into the following four grades: absent, mild (centrilobular involvement limited to one-third of the lobular area), moderate (centrilobular involvement extending in two-thirds of the lobular area), and severe (complete lobular involvement or centrilobular involvement extending to adjacent lobules with bridging congestion). Centrilobular or venular fibrosis was classified into the following three grades: absent, mild (<50% of veins and sinusoids evaluated in 20 fields at ×200 magnification), and moderate (>50% of veins and sinusoids evaluated in 20 fields at ×200 magnification). Nodular transformation was classified into the following four grades: absent, mild (focal occasionally distinct nodular hyperplasia), moderate (focal distinct nodular hyperplasia), and severe (diffuse nodular hyperplasia corresponding to nodular regenerative hyperplasia [NRH]). Steatosis was classified into the following four grades: absent, mild (steatosis in 10–30% of hepatocytes), moderate (steatosis in 30–60% of hepatocytes), and severe (steatosis in >60% of hepatocytes). Peliosis and hepatocellular damage were described as present or absent.

**Establishment and maintenance of patient-derived liver organoids**

Fresh tissue samples obtained as residual tissue during the surgery were minced fresh into small pieces, at less than 0.5 mm in diameter, washed three times with ice-cold wash solution (HBSS supplemented with 1% FBS and 1% penicillin/streptomycin [P/S]), and subsequently digested with 2.5 mg/mL Liberase (Merck, 05401160001) and 10 μg/mL DNase I (Merck, DN25) for 60 to 90 min at 37°C. After pipetting, the fragments were passed through a 100 μm strainer (pluriSelect Life Science) and collected. When the fragments were observed to be larger than 100 μm, they were additionally treated with 5 mL of TrypLE Express (Thermo Fisher Scientific, 12604013) containing 10 μg/mL DNase I at 37°C for 10 to 15 min. The digests were collected and centrifuged at 400×*g* for 5 min at 4°C. The resulting pellets were washed with 1 mL of wash solution, centrifuged at 400×*g* for 5 min at 4°C, suspended in Matrigel (Corning, 356231), seeded in 24-well culture plates at 30 μL each, and cultured as described by Huch *et al.*^6^ and Broutier *et al*.^7^ In brief explanation, expansion medium was Advanced DMEM/F-12 (Thermo Fisher Scientific, 12634028) supplemented with 1% P/S (Thermo Fisher Scientific, 15140122), 1% GlutaMAX (Thermo Fisher Scientific, 12604013), 10 mM HEPES (Thermo Fisher Scientific, 15630080), 1:50 B27 supplement (Thermo Fisher Scientific, 12587010) and 1:100 N2 supplement (Thermo Fisher Scientific, 17502048), 1 mM *N*-Acetyl-L-cysteine (NAC, FUJIFILM Wako Pure Chemical, 015-05132), 10 nM human gastrin I (Merck, G9145) and the growth factors: 50 ng/mL human EGF (Peprotech, AF-100-15), 10% (vol/vol) Rspo1-conditioned medium (homemade prepared from 3710-001-01; R&D systems), 100 ng/mL human FGF10 (Peprotech, AF-100-26), 25 ng/mL human HGF (Peprotech, 100-39), 10 mM Nicotinamide (Sigma-Aldrich, N0636), 5 μM A83-01 (Tocris Bioscience, 2939), and 10 μM Forskolin (FUJIFILM Wako Pure Chemical, 067-02191). For the first 3–7 days of culture, the medium was supplemented with 25 ng/mL human Noggin (Peprotech, 120-10C), 30% (v/v) Wnt3a-conditioned medium (homemade prepared from CRL-2647; ATCC), and 10 μM Y-27632 (FUJIFILM Wako Pure Chemical, 036-24023). After 7–14 days, liver organoids were harvested and seeded for the next passage in Matrigel or Basement Membrane Extract, Type 2 (R&D Systems, 3533-010-02).

For the morphological evaluation, liver organoids were fixed in a 10 % formalin neutral buffer solution (FUJIFILM Wako Pure Chemical), embedded in paraffin, and sectioned at 2 μm for H&E staining.

**Hepatocyte differentiation culture**

To obtain liver organoids that differentiated into hepatocytes, established organoids were maintained in an expansion medium supplemented with 25 ng/mL human BMP-7 (R&D Systems, 354-BP-010) for 3–5 days after the last passage and then cultured for 5–9 days in glucose-based hepatocyte differentiation medium (standard differentiation medium). To examine the effects of galactose in the culture medium, liver organoids were additionally cultured for 3 days in a galactose-based hepatocyte differentiation medium (galactose differentiation medium) or standard differentiation medium. Hepatocyte differentiation medium contains glucose-free DMEM/Ham’s F-12 (Nacalai Tesque, 09893-05) supplemented with 17 mM glucose (FUJIFILM Wako Pure Chemical, 049-31165) or 17 mM galactose (Nacalai Tesque, 16511-62), 1% P/S, 10 mM HEPES, 0.5 mM sodium pyruvate solution (Merck, S8636), 400 mg/L AlbuMAX II (Thermo Fisher Scientific, 11021029), 7.5 mg/L Human transferrin (Nacalai Tesque, 34443-44), 10 mg/L human insulin (Nacalai Tesque, 12878-86), 1.52 mg/L L-Ascorbic Acid (Merck, A92902) and the supplementals described in Huch *et al*.^6^ and Broutier *et al.*^7^; 1:50 B27 supplement, 1:100 N2 supplement, 1 mM NAC, 10 nM human gastrin I, 50 ng/mL human EGF, 25 ng/mL human HGF, 100 ng/mL human FGF19 (Peprotech, 100-32), 10 μM DAPT (Merck, D5942), 3 μM dexamethasone (FUJIFILM Wako Pure Chemical, 041-18861), 0.5 μM A83-01, and 25 ng/mL human BMP7.

**RNA isolation and quantitative Real-time PCR (qPCR) Analysis**

RNA was isolated from freshly resected liver tissue or liver organoids using the RNeasy Mini Kit (QIAGEN) or RNeasy Micro Kit (QIAGEN), respectively, according to the manufacturer’s protocol. cDNA was generated using the harvested RNA as the template with Super Script IV VILO Master Mix (Thermo Fisher Scientific). For each sample, the PCR reaction mix was prepared in a total volume of 20 μL containing 2X TaqPath qPCR Master Mix, CG (Thermo Fisher Scientific), 20X TaqMan Expression Assay probes (Thermo Fisher Scientific), and cDNA template. TaqMan Expression Assay probes used the following genes: *GAPDH* (4326317E), *ALB* (Hs00609411_m1), *CYP3A4* (Hs00604506_m1), *LGR5* (Hs00969422_m1), and *HNF4A* (Hs00230853_m1). qPCR was performed in three wells of a StepOne Real-Time PCR System (Thermo Fisher Scientific) or a QuantStudio3 Real-Time PCR System (Thermo Fisher Scientific). The results were expressed as the average fold change in gene expression calculated using the 2^-ΔΔCt^ method using *GAPDH* as the internal control.

**Mitochondrial membrane potential assay**

Differentiated liver organoids were cultured with or without L-OHP treatment to determine the mitochondrial membrane potential. For the L-OHP treatment, the culture medium was replaced with fresh L-OHP-containing medium (NAC-free) every 24 h for up to 48 h. For the CCCP treatment, liver organoids were cultured in galactose differentiation medium (NAC-free) containing 100 μΜ CCCP (abcam, ab141229) for 1.5 h. Liver organoids were collected from the embedded gel by gently pipetting up and down using ice-cold wash buffer (HBSS supplemented with 0.25% BSA and 1% P/S) and incubated at 37℃ in a fully humidified 5% CO_2_ atmosphere for 30 min. The mitochondrial membrane potential was determined using the JC-1 MitoMP Detection Kit (Dojindo) according to the manufacturer’s protocol. Fluorescence images of liver organoids were acquired using a ZEISS LSM900 system with ZEISS ZEN3 (blue edition) software. The fluorescence intensity was measured using a BZ-X810 microscope with BZ-H4C (Keyence).

**Mitochondrial oxidative stress assay**

Differentiated liver organoids were collected from the embedded gel by gently pipetting up and down using ice-cold wash buffer (HBSS supplemented with 0.25% BSA and 1% P/S) and then cultured in galactose differentiation medium (NAC-free) containing L-OHP. After 2 h of culture, liver organoids were incubated at 37℃ in a fully humidified 5% CO_2_ atmosphere for 10 min with mtSOX Deep Red (Dojindo) 10 μM working solution, and the working solution was then replaced with HBSS. Bright-field and fluorescence images were acquired using a BZ-X810 microscope to identify cells emitting red fluorescence with mitochondrial superoxide (O_2_^●-^). The cross-sectional area and fluorescence intensity were measured using the BZ-H4C application.

**ATP content assay**

Liver organoids were differentiated into hepatocytes in 96-well plates, as described for the LDH leakage assay. The assay was performed in triplicate. For oligomycin A treatment, liver organoids were cultured with 5 μΜ oligomycin A (AdipoGen Life Sciences, AG-CN2-0517) for 2 h. For L-OHP treatment, liver organoids were cultured in a galactose differentiation medium containing L-OHP at concentrations of 0, 20, 40, 80, 160, and 320 μM for 72 h. Intracellular ATP content was measured by luminescence using the CellTiter-Glo Luminescent Cell Viability Assay (Promega). Luminescence was measured using a Multimode Microplate Reader Infinite M200 Plex (Tecan).

**Lactate production assay**

Lactate production by the differentiated liver organoids was determined in triplicate. The culture medium was replaced with fresh medium and the cells were collected within 24 h for analysis. Lactate production was determined by measuring the amount of lactate in the supernatant, using a lactate assay kit (Dojindo). Absorbance was measured at 450 nm using a Multimode Microplate Reader Infinite M200 Plex.

**Glutathione (GSH) Assay**

Liver organoids were differentiated and treated with L-OHP in triplicate, as described for the LDH leakage assay. Total GSH was measured using the GSH-Glo Glutathione assay (Promega). Luminescence was measured using a Multimode Microplate Reader Infinite M200 Plex.

**References**

1 Prassopoulos P, Daskalogiannaki M, Raissaki M, Hatjidakis A, Gourtsoyiannis N. Determination of normal splenic volume on computed tomography in relation to age, gender and body habitus. *Eur Radiol*. 1997; 7: 246-248.

2 Rubbia-Brandt L, Audard V, Sartoretti P, et al. Severe hepatic sinusoidal obstruction associated with oxaliplatin-based chemotherapy in patients with metastatic colorectal cancer. *Ann Oncol*. 2004; 15: 460-466.

3 Rubbia-Brandt L, Lauwers GY, Wang H, et al. Sinusoidal obstruction syndrome and nodular regenerative hyperplasia are frequent oxaliplatin-associated liver lesions and partially prevented by bevacizumab in patients with hepatic colorectal metastasis. *Histopathology*. 2010; 56: 430-439.

4 Ryan P, Nanji S, Pollett A, et al. Chemotherapy-induced liver injury in metastatic colorectal cancer: semiquantitative histologic analysis of 334 resected liver specimens shows that vascular injury but not steatohepatitis is associated with preoperative chemotherapy. *Am J Surg Pathol*. 2010; 34: 784-791.

5 Nalbantoglu IL, Tan BR, Jr., Linehan DC, Gao F, Brunt EM. Histological features and severity of oxaliplatin-induced liver injury and clinical associations. *J Dig Dis*. 2014; 15: 553-560.

6 Huch M, Gehart H, van Boxtel R, et al. Long-term culture of genome-stable bipotent stem cells from adult human liver. *Cell*. 2015; 160: 299-312.

7 Broutier L, Andersson-Rolf A, Hindley CJ, et al. Culture and establishment of self-renewing human and mouse adult liver and pancreas 3D organoids and their genetic manipulation. *Nat Protoc*. 2016; 11: 1724-1743.
