# Supplementary material for: Prediction for oxaliplatin‐induced liver injury using patient‐derived liver organoids
Source: Cancer Med. 2024 Feb 24;13(3):e7042. doi: 10.1002/cam4.7042 (PMC10891453; doi:10.1002/cam4.7042)
Supplement: Supplementary file 2 — Figures S1–S4. [file CAM4-13-e7042-s001.pdf]

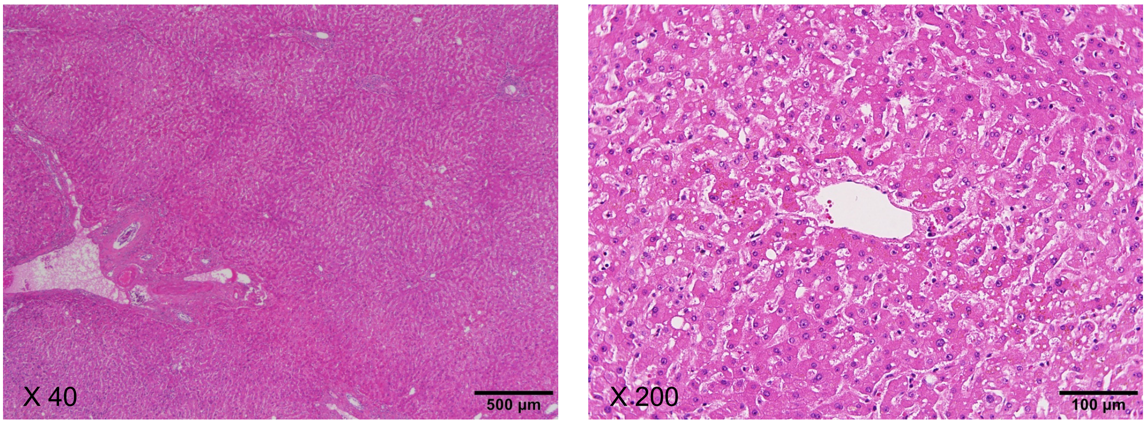

Figure S1. Histology of liver tissue of LM23 in the low-grade liver injury group. Representative Victoria Blue (VB)-H&E staining images of non-cancerous liver tissue treated with L-OHP-based chemotherapy. A low-power field is shown in the left panel. A high-power field is shown in the right panel.

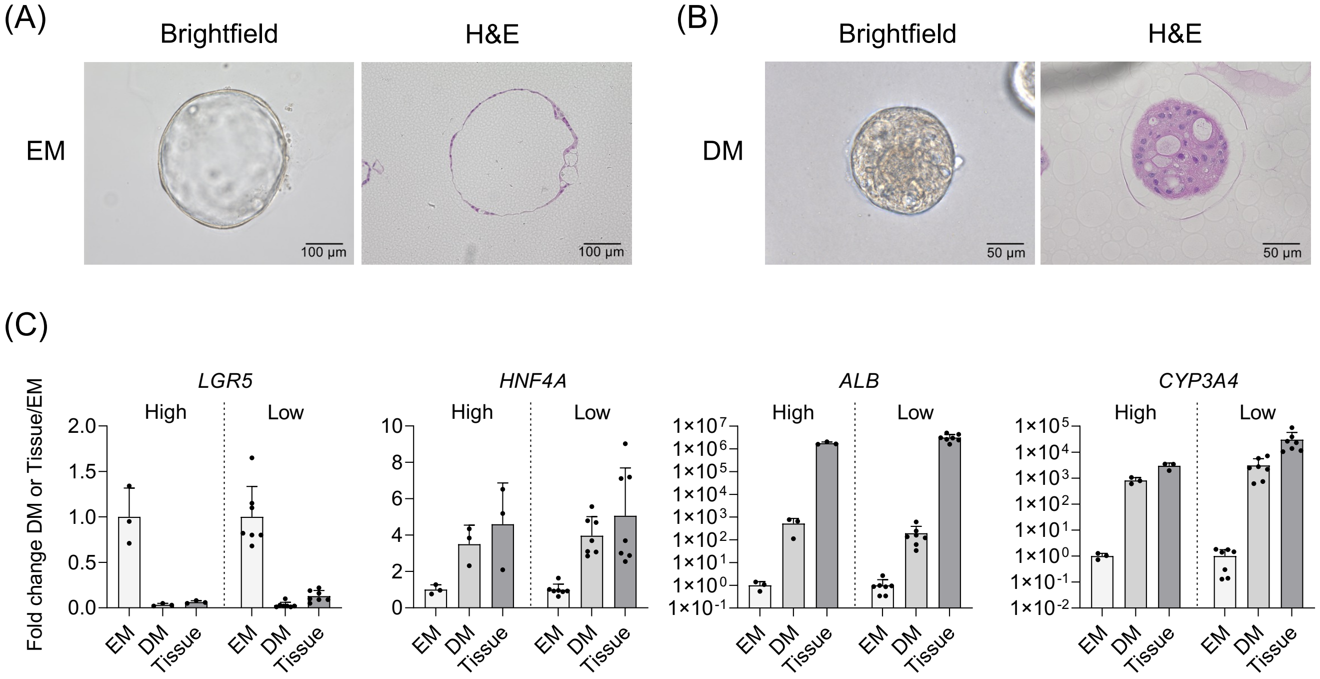

Figure S2. Characterization of liver organoids established from non-cancerous liver tissues.

(A, B) Representative bright-field and H&E staining images of a liver organoid cultured in expansion medium (EM) (A) and in differentiation medium (DM) (B). (C) Fold changes in the expression levels of the indicated genes in liver organoids cultured in conditioned media (EM and DM) and in non-cancerous liver tissues (Tissue). Each dot indicates independent patients in the high- or low-grade groups. Bars indicate mean ± SD.

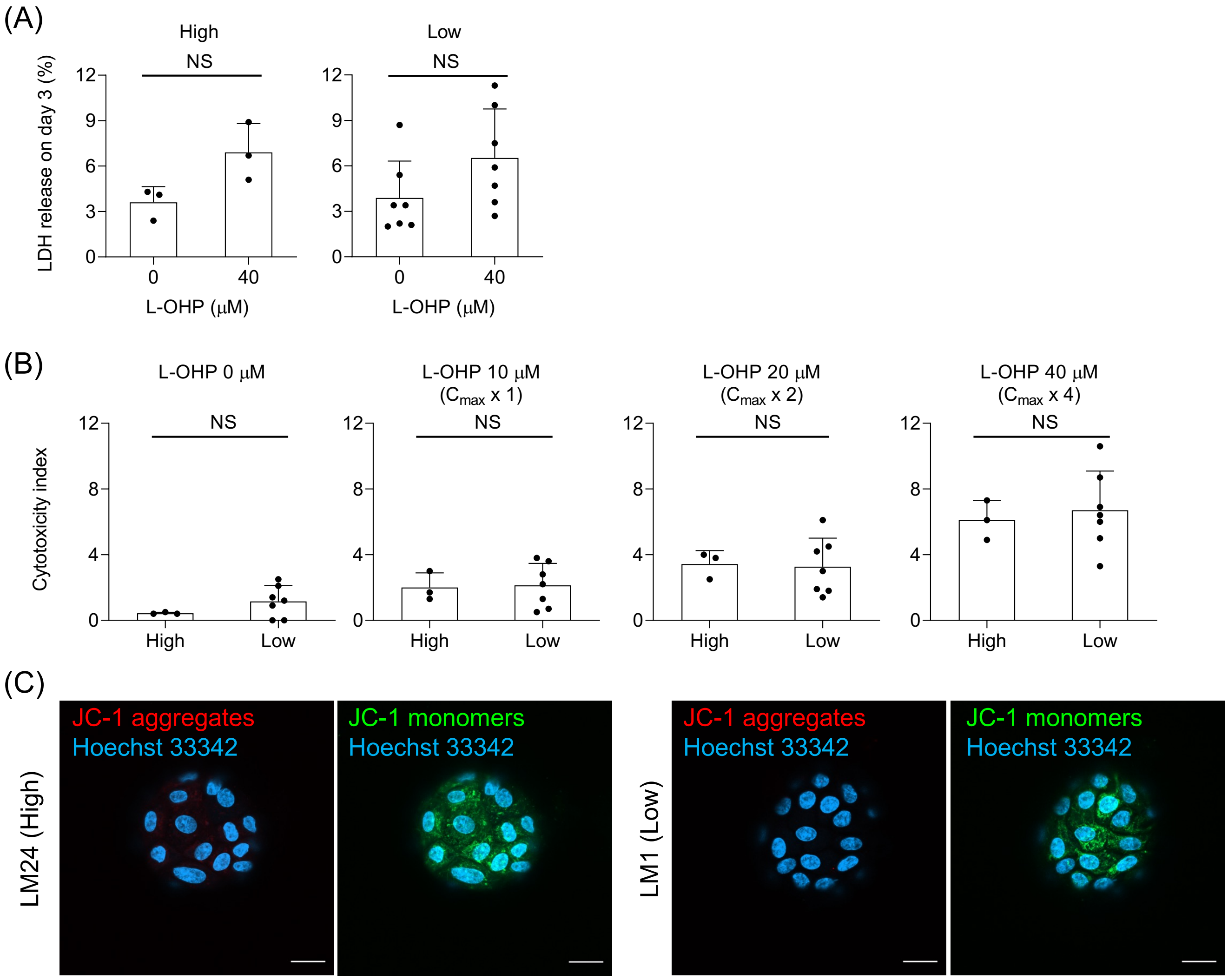

Figure S3. Assessment of cytotoxicity in liver organoids cultured in standard differentiation medium.

(A-C) Liver organoids were cultured in a standard differentiation medium for 8–11 days. (A) LDH release from liver organoids on day 3 of repeated doses of L-OHP. (B) Cytotoxicity index of liver organoids treated with repeated doses of L-OHP at the indicated concentrations for 72 h. (C) Mitochondrial condition was evaluated with a JC-1 probe. Representative images of JC-1-stained liver organoids are shown. Red, JC-1 aggregates; green, JC-1 monomers; blue, Hoechst 33342 (nuclei). Scale bar, 20 μm. For all bar graphs, each dot indicates independent patients in the high- or low-grade groups. Bars indicate mean ± SD. Statical significance was determined with a two-sided Welch's *t*-test (A) or two-sided Mann–Whitney *U* test (B). NS, Not Significant.

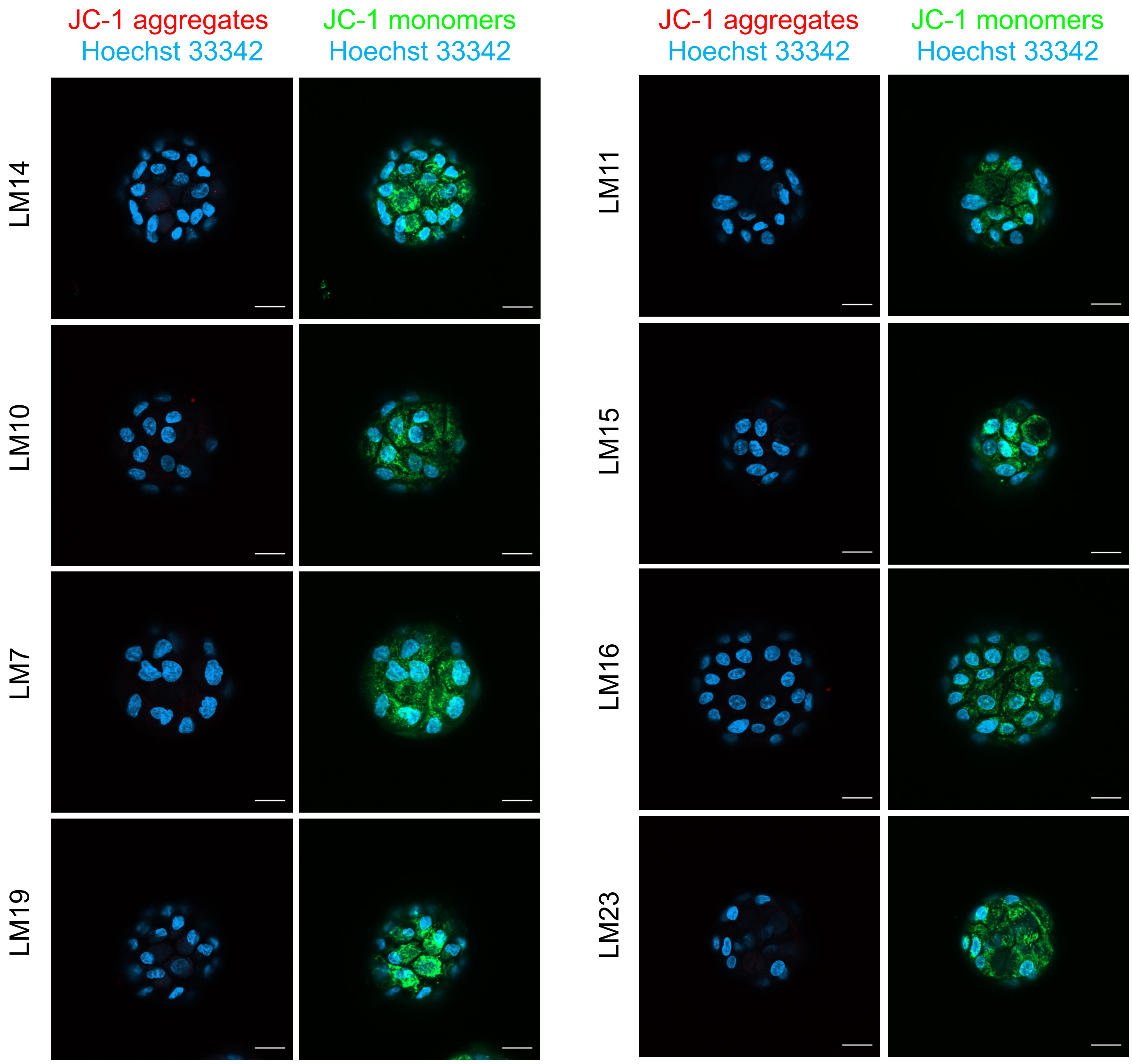

Figure S4. Representative images of JC-1-stained liver organoids cultured in standard differentiation medium.

The mitochondrial condition of liver organoids from each patient was evaluated using a JC-1 probe. Representative images of the JC-1-stained liver organoids. Red, JC-1 aggregates; green, JC-1 monomers; blue, Hoechst 33342 (nuclei). Scale bar, 20 μm.
