# Supplementary material for: Prediction for oxaliplatin‐induced liver injury using patient‐derived liver organoids
Source: Cancer Med. 2024 Feb 24;13(3):e7042. doi: 10.1002/cam4.7042 (PMC10891453; doi:10.1002/cam4.7042)
Supplement: Supplementary file 3 — Table S1. [file CAM4-13-e7042-s002.docx]

**Supplementary Table S1 Histopathological findings of liver tissues of the patients**

|  | **High-grade group** | | | | **Low-grade group** | | | | | | | |
| --- | --- | --- | --- | --- | --- | --- | --- | --- | --- | --- | --- | --- |
|  |  |  |  |  | |  | |  |  |  |  |  |
|  | **LM24** | **LM14** | **LM10** | | **LM7** | | **LM19** | **LM1** | **LM11** | **LM15** | **LM16** | **LM23** |
| Sinusoidal dilatation | Mild | Moderate  to severe | Moderate | | Mild | | Mild | Absent | Moderate | Absent | Moderate | Absent |
| Centrilobular/  venular fibrosis | Mild | Absent | Mild | | Absent | | Mild | Absent | Absent | Absent | Mild | Absent |
| Nodular transformation | Mild | Mild | Moderate | | Absent | | Absent | Absent | Mild | Absent | Mild | Absent |
| Peliosis | Absent | Absent | Absent | | Absent | | Absent | Absent | Absent | Absent | Absent | Absent |
| Hepatocellular damage | Present | Present | Present | | Absent | | Absent | Absent | Absent | Absent | Absent | Absent |
| Steatosis | Absent | Absent | Moderate | | Mild | | Moderate | Mild | Absent | Mild | Severe | Absent |
